# Maplaria: a user friendly web-application for spatio-temporal malaria prevalence mapping

**DOI:** 10.1186/s12936-021-04011-7

**Published:** 2021-12-20

**Authors:** Emanuele Giorgi, Peter M. Macharia, Jack Woodmansey, Robert W. Snow, Barry Rowlingson

**Affiliations:** 1 Centre for Health Informatics, Computing, and Statistics, Lancaster Medical School, Lancaster University, Lancaster, UK; 2 Population Health Unit, KEMRI-Wellcome Trust Research Programme, Nairobi, Kenya; 3 Centre for Tropical Medicine and Global Health, Nuffield Department of Medicine, University of Oxford, Oxford, UK

**Keywords:** Malaria, Model based geostatistics, National malaria control programme, Web application, Sub Saharan Africa, Malaria mapping, Cross-sectional surveys

## Abstract

**Background:**

Model-based geostatistical (MBG) methods have been extensively used to map malaria risk using community survey data in low-resource settings where disease registries are incomplete or non-existent. However, the wider adoption of MBG methods by national control programmes to inform health policy decisions is hindered by the lack of advanced statistical expertise and suitable computational equipment. Here, *Maplaria,* an interactive, user-friendly web-application that allows users to upload their own malaria prevalence data and carry out geostatistical prediction of annual malaria prevalence at any desired spatial scale, is introduced.

**Methods:**

In the design of the Maplaria web application, two main criteria were considered: the application should be able to classify subnational divisions into the most likely endemicity levels; the web application should allow only minimal input from the user in the set-up of the geostatistical inference process. To achieve this, the process of fitting and validating the geostatistical models is carried out by statistical experts using publicly available malaria survey data from the Harvard database. The stage of geostatistical prediction is entirely user-driven and allows the user to upload malaria data, as well as vector data that define the administrative boundaries for the generation of spatially aggregated inferences.

**Results:**

The process of data uploading and processing is split into a series of steps spread across screens through the progressive disclosure technique that prevents the user being immediately overwhelmed by the length of the form. Each of these is illustrated using a data set from the Malaria Indicator carried out in Tanzania in 2017 as an example.

**Conclusions:**

Maplaria application provides a user-friendly solution to the problem making geostatistical methods more accessible to users that have not undertaken formal training in statistics. The application is a useful tool that can be used to foster ownership, among policy makers, of disease risk maps and promote better use of data for decision-making in low resource settings.

**Supplementary Information:**

The online version contains supplementary material available at 10.1186/s12936-021-04011-7.

## Background

Across malaria endemic countries in sub-Saharan Africa (SSA), national malaria control programmes (NMCPs) develop guidelines, strategies, and policies that are adapted in accordance to local malaria endemicity levels [1]. Following guidance from the Global technical strategy for malaria 2016–2030 [2], NCMPs are required to stratify their countries sub-nationally based on endemicity, so as to realign their malaria control activities [3, 4]. For example, regions with prevalence lower than 1% may need to adapt their strategies for malaria elimination [5], whilst areas with a prevalence above 30% may still require intensive and sustained vector control [3]. To this end, NMCPs often require estimates of prevalence to be aggregated over geographical units used for decision-making, such as districts, regions or counties. In addition to this, NCMPs also carry out assessment of historical malaria risk for monitoring, evaluation and comparison with the receptive risk [6]. Disease risk mapping is thus essential in providing timely and reliable information on the spatial heterogeneity of malaria endemicity in a country and how this changes over time.

The disease risk mapping process is carried through the analysis of data from cross-sectional surveys, such as malaria indicator surveys (MIS), demographic and health surveys (DHS), school surveys and other non-routine surveys. Over the last decade, model-based geostatistical (MBG) methods have become an established set of modern statistical tools for interpolating malaria risk within a geographical area of interest using cross-sectional survey data [7, 8]. As a result, risk maps generated from MBG models have been increasingly adopted by NMCPs in SSA, where the burden remains high relative to other regions, to inform policy decisions for monitoring, evaluation and to inform health policies [9, 10].

Despite this, one of the major obstacles to the wider application of MBG methods by NMCPs is that they require specialized statistical expertise, as well as advanced computer programming skills in the R, WINBUGS or stan software environments [11–13]. Due to the lack of such resources, especially in many of the malaria-endemic countries, there is a risk that policy decisions are made using sub-optimal approaches that do not handle the uncertainty in a statistically rigorous fashion. For example, policy makers often base their decisions on the exceedance of prevalence thresholds using subnational empirical prevalence by ignoring the uncertainty inherent to these, which can lead to misclassifications of the subnational evaluation units [3, 14]. This issue has been partly alleviated by outsourcing statistical expertise to mostly Western research institutes (for example, Malaria Atlas Project—MAP and the Institute of Health Measure Evaluation—IHME), which have provided global maps of malaria prevalence generated using state-of-the-art MBG models [15, 16]. However, policy makers have raised concerns about the breadth and granularity of data in externally implemented geostatistical models and expressed interest in having direct control by participating in all stages of malaria risk mapping [10], in order to promote ownership and use of malaria maps that are used in the decision making process.

To address these issues, in this paper, a user-friendly web application, called Maplaria (https://fhm-chicas-Maplaria.lancs.ac.uk), for malaria prevalence mapping, aimed at users who possess only limited statistical knowledge is introduced. Maplaria allows the user to upload their own data and carry out prediction of annual malaria prevalence based on geostatistical models that are fitted to and validated using publicly available data on malaria surveys conducted in SSA from Population Health Dataverse [17] with data that are uploaded by the user. Outside the field of malaria epidemiology, several user-friendly web applications have been developed to promote the use of spatial statistical methods for disease mapping and overcome the requirement for advanced programming and statistical skills. Of note among these are the open-source web application created by Tomlinson et al. (2019) known as MaDD (Malaria Data by District) which allows visualisation of key malaria risk maps from the MAP at a local level for decision makers [18], and, in the context of small area estimation, SpatialEpiApp [19], and SSTCDapp [20].

However, there are currently no user-friendly applications that implement spatio-temporal geostatistical models for malaria prevalence mapping and allow users to generate predictive maps of malaria prevalence using their own cross-sectional data. The web application presented in this paper, Maplaria, allows the user to perform spatio-temporal geostatistical prediction of annual *Plasmodium falciparum* prevalence among children aged 2 to 10 years (*Pf*PR_2-10_), in a given country within SSA. In the next sections, the following are introduced, the principles that have been adopted for developing Maplaria, the structure of the web-application from a software engineer perspective and an illustration using data from a Malaria Indicator Survey (MIS) conducted in Tanzania in 2017.

## Methods

### Criteria for developing a user-friendly interface for malaria mapping

The intended end-users of Maplaria are public health staff from NMCPs, including researchers who may have undertaken only limited training in statistics. The requirements for using Maplaria effectively are a good understanding of basic descriptive statistics and primary GIS data models, in particular raster and vector data models.

To make advanced statistical methodology more accessible to this kind of end-user, two main criteria have been considered throughout the development of Maplaria: C1) the web-application should be used to answer a well-defined health policy question; C2) the web-application should automate geostatistical spatio-temporal prediction while requiring minimal input from the user. In considering C1, Maplaria has been primarily developed with the goal of providing predictive inferences on the likelihood of exceeding policy-relevant prevalence thresholds at any desired spatial scale, such as villages or subnational administrative units. These thresholds are important to develop targeted malaria control interventions by taking into account the heterogeneity of malaria transmission intensity [3, 5, 14, 21].

To pursue C2, fitting and validation of a geostatistical model should be first carried out by geostatistical experts using an initial set of publicly available data henceforth referred to as *baseline* data; more details on the baseline data are provided in the next section. The resulting model, developed for a single country, is then uploaded onto Maplaria which then allows users to upload new data that are incorporated with the *baseline* data to generate updated maps for any desired year. As result of this approach, the functionalities implemented in Maplaria only focus on the set-up of geostatistical prediction, whilst model fitting and validation is carried out outside of the web-application without necessarily requiring any input from the Maplaria user.

**Fig. 1:**
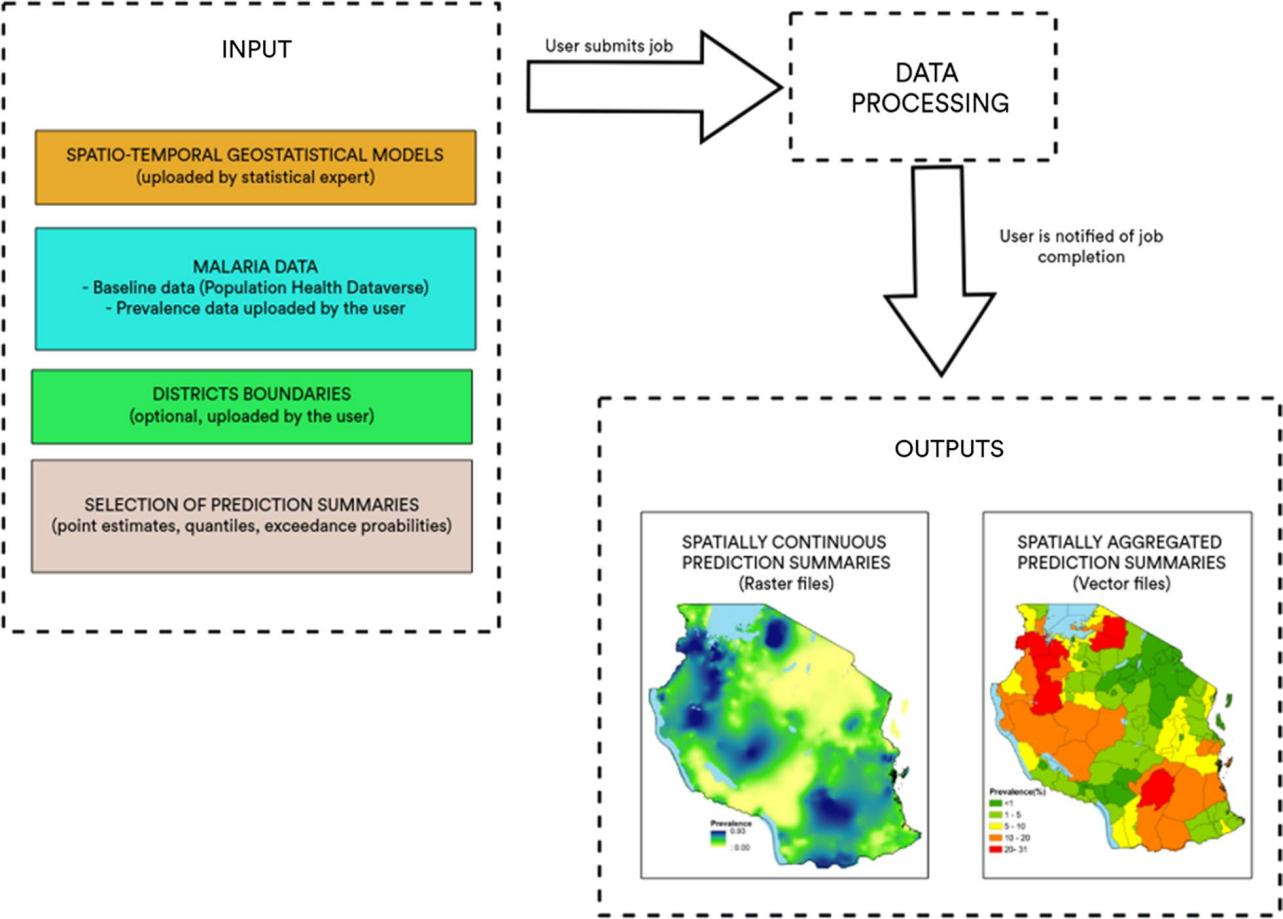
Diagram of the Maplaria web-application, with summary of input and output data

Following these criteria, Maplaria application was designed as shown in Fig. 1. Among the input, two categories can be distinguished: those that are provided by the geostatistical expert, and those by the user. The data uploaded by the user consists of a malaria prevalence data-set which is then combined with the *baseline* data. The combined data-set is then processed by Maplaria to generate a predictive surface either on a spatially continuous or aggregated format for a given year specified by the user. The baseline data can be visualized in the web-app and directly downloaded from Population Health Dataverse [17]. More details on the architecture of Maplaria are provided in the “Results'' section.

### Malaria survey data for set-up of geostatistical models in Maplaria

The *baseline* data that are used to implement the geostatistical models in *Maplaria* are publicly available malaria community survey data consisting of 50,424 parasite prevalence surveys which range from 1900 to 2015 for 43 countries in SSA. In Maplaria, a subset consisting of all the malaria survey data from 2000 thereafter is extracted for a specific SSA country in order to fit a country-specific geostatistical model. This approach is used in order to tailor the parameter estimates of the geostatistical model to data more recently collected and avoid the need for a more complex modelling approach that may be required when analyzing data on large spatial and temporal scales. The baseline data was sourced from electronic data searches of peer-reviewed publications, and opportunistic sources where malaria scientists, research institutes, government and non-government agencies were contacted to provide data. The key variables contained in the data are summarized in Table 1. Detailed descriptions of data assembly are available at Snow et al., 2017 while the datasets are publicly available from the Population Health Dataverse [17, 22].

**Table 1:** Names and description of the variables of the malaria prevalence data from Harvard Dataverse [17]

Variable	Description
ID	Record ID
Country	Country where the data was collected
Afr Admin 2 Code	Administrative division number/code within a country
Afr Admin name	Name of administrative division within a country
Area Type	Either point (villages, schools and communities < 5km^2^), polygon (large administrative polygons) or wide area (areas > 5 km^2^)
Lat	Latitude of the surveyed community
Long	Longitude of the surveyed community
MM	Month when the survey was conducted
YY	Year when the survey was conducted
LoAge	Lowest age in surveyed community
UpAge	Highest age in surveyed community
Ex	Total number of those examined
Pf	Positive of *Plasmodium falciparum* parasite
*Pf*PR_2-10_	*Plasmodium falciparum* parasite rate in children aged 2–10 years
Method	Method used to detect parasite

### Geostatistical modelling of the malaria survey data from the Harvard data-base

The geostatistical models that are fitted to the assembled community malaria surveys from the Harvard database are described in detail elsewhere [3, 8, 14]. In this section, the model formulation of the geostatistical models that are implemented in Maplaria at the time of writing are presented. Importantly, the model specifications may change in the future, with updates posted at the methods page of Maplaria: https://fhm-chicas-Maplaria.lancs.ac.uk/page/methods.

Specifically $Y_{i}$, conditionally on $S\left( {x_{i} ,t_{i} } \right)$ and $Z\left( {x_{i} ,t_{i} } \right)$, are assumed to be mutually independent Binomial variables with probability of a positive test $\left( {x_{i} , t_{i} } \right)$ with logit-linear predictor given as:

$$log\left\{ {\frac{{p\left( {x_{i} ,t_{i} } \right)}}{{1 - p\left( {x_{i} ,t_{i} } \right)}}} \right\} =\,\alpha + \beta_{1} a_{i}^{m} + \beta_{2} a_{i}^{M} + \gamma d_{i} + S\left( {x_{i} ,t_{i} } \right) + Z\left( {x_{i} ,t_{i} } \right)$$

where $S\left( {x_{i} ,t_{i} } \right)$ and $Z\left( {x_{i} ,t_{i} } \right)$ are random effects that account for unexplained spatio-temporal and unstructured variation in prevalence, respectively, due to unmeasured malaria risk factors. The spatial process *S*(*x*, *t*) is assumed to be stationary and isotropic with space–time covariance function given as:

$${\text{cov}} \{ S(x,t),S(x^{\prime},t^{\prime})\} = \sigma^2\frac{1}{(1 + |t - t|/\psi )}\exp \{ - ||x - x^{\prime}||/\phi \}$$

where: *σ*^2^ is the variance of *S*(*x*,*t*); |*t* − *t*| is the time separation, in years, between observations; ||*x* − *x*′|| is the Euclidean distance in space, measured in km, between observations; *Ψ* is a scale parameter that regulates how fast the temporal correlation decays to zero for increasing |*t* − *t*|; and, *ϕ* is a scale parameter that regulates how fast the spatial correlation decays to zero for increasing ||*x* − *x*′||. *τ*^2^ is used to denote the variance of the $Z_{i}$, the variation within communities (i.e. genetic and behavioural traits). The covariates $a_{i}^{m}$ and $a_{i}^{M}$ are the minimum and maximum age among the sampled individuals at a location $x_{i}$ and year $t_{i}$. In carrying out the spatio-temporal predictions, $a_{{}}^{m}$ and $a_{{}}^{M}$ are set to 2 and 10 years, respectively, so as to standardize to a single age range of 2–10 years conventionally used for malaria risk mapping [23]. Finally, the variable $d_{i}$ is set to be an indicator variable indicating whether a rapid diagnostic test ($d_{i} = 1$) or microscopy test ($d_{i} = 0$) has been to diagnose the sampled individuals with malaria. The estimation of the parameters of the geostatistical models is carried using the Monte Carlo maximum likelihood method implemented in the PrevMap R package [24].

To quantify the likelihood of exceeding a predefined prevalence threshold *l*, Maplaria allows the user to specify multiple values for *l* and computes the exceedance probability (EP) resulting from the fitted country-specific geostatistical model. Maplaria allows any threshold value to be defined. EPs are summary of the uncertainty on the exceedance or not of prevalence thresholds that are interpreted as follows: values close to 100% indicates that *Pf*PR_2-10_ is highly likely to be above the threshold l; if close to 0%, *Pf*PR_2-10_ is highly likely to be above the threshold l; if close to 50%, *Pf*PR_2-10_, is equally likely to be above or below the threshold l, hence corresponding to a high level of uncertainty.

## Results

### Accessing Maplaria

Maplaria can be accessed at the link https://fhm-chicas-Maplaria.lancs.ac.uk/ using any major web browser. The welcome page has a menu with three options which include applying for an account, signing in for existing account holders and accessing a demo account to explore the features of the web application. The interface of Maplaria consists of the following tabs.

#### My datasets

The processing status of submitted data to Maplaria for generating predictions of prevalence.

#### Upload dataset

This is the main tab that allows the user to upload new data and set the parameters for running the geostatistical models and generate the spatio-temporal predictions for prevalence. The user has the option of giving consent to make their uploaded data publicly available but, otherwise, these are hidden to other users of the application, including the Maplaria management team.

#### Public datasets

In this tab, users can access and visualize data with their associated prevalence predictions that have been made publicly available by the owners and existing Maplaria users.

#### Who we are

Short biographies of the creators of Maplaria.

#### Methodology

Here, references for the underpinning methodology of Maplaria are provided. In addition, a summary report for each country implemented in Maplaria is provided, with details on the geostatistical model formulation and the parameter estimates that are used to generate the spatio-temporal predictions for prevalence.

#### About

A short description of what Maplaria is and should be used for.

#### FAQ

Answers to frequently asked questions that have been raised by Maplaria users.

#### Tutorial

Here, users can find animated tutorials on how to run Maplaria, starting from the uploading data to the visualization of the results.

#### Contact

A form for queries about Maplaria and raising issues arising from the use of the functionalities in the “Upload dataset” tab.

The demo account provides access to all the features of Maplaria, as listed above, except for “My datasets” and “Upload dataset”.

### Uploading and processing of user’s data in Maplaria

The uploading and processing of data is summarized in the architecture diagrams of Additional file 1: Figure S1 and Additional file 2: Figure S2, in the supplementary material. The process has been split into a series of steps spread across screens through the progressive disclosure technique that prevents the user being immediately overwhelmed by the length of the form. Steps are hidden (masked) or added based on the previous choices and selection made by the user. Users can return to the previous screen to make changes, without losing any inputted data.

#### Step 1

Users select the country for which mapping is to be carried out, from a list of countries for which they have been granted access. First users of Maplaria have access to a single country for which they have been granted access at the time of creation of their account. Access to more than one country can be requested by email to the Maplaria management team*.*

#### Step 2

In the second step the user is required to upload survey data, in .csv, .xlsx, or .tab formats. The user is required to format columns with specific column names. The user will be shown an error if any of the required columns for survey data are missing or incorrectly formatted, or if there are duplicate entries for the same location for the same year.

#### Step 3

The user must select the input arguments. This includes the year for predictions, and choosing between spatially continuous (raster file), aggregated (estimates provided at administrative regions), or both. Users also select settings related to which outputs they need including point estimates, standard errors, quantiles, and thresholds. At least one option must be selected. Maplaria only allows submission of jobs for a single year for which predictions are required. Hence, if predictions are required for multiple years, multiple jobs should be submitted by the user for every year.

#### Step 4

If the user has selected spatially continuous predictions, they are now required to select whether they want to draw a boundary, upload a shape file, or skip. Selecting "draw" displays a map with OpenStreetMap tiles, and the user can click on it to draw a polygon over their desired area. Selecting "upload" requires the user to upload a shape file, and will be useful if the user wants a precisely defined area from data outside of Maplaria. The user can also skip drawing or uploading, if they choose to "skip" then Maplaria will use the national borders of the chosen country to create a regular grid for the spatial predictions. Next, if the user selected aggregated predictions, they must upload a shape file and enter a column name that can be used to index each boundary contained in the shape file.

#### Step 5

Before submitting the user is shown a preview of all the data they have uploaded on a map, and given a chance to go back to make any corrections. The user can then submit their dataset, which is marked as ready for processing.

The Maplaria API server stores all uploaded user data and is responsible for managing the execution of dataset processing. At a regular interval, currently set at two minutes, the database is queried to locate all datasets awaiting processing and the oldest input is selected from the queue. The arguments for this dataset, including the types of predictions, year for predictions, and locations of survey data and boundary files, are written locally to a JSON file. Next, the API server spawns a daemon process to execute the Rscript which contains the algorithm for carrying out geostatistical prediction. The location of this JSON file is passed through standard input to the Rscript, and the Rscript is responsible for reading the contents of this JSON file during processing. The process ID is recorded in the database against the dataset so that it can be queried later. This process repeats until there are no more datasets left in the queue.

The server also queries the database for all currently executing datasets. Each of these is checked to see if a process is still executing for that dataset's process ID. If it is still executing, then this dataset is ignored for now, otherwise the output files are read. If the actual output matches the expected output, that is all the files are available for the given input arguments, then it is considered successful and the output data is parsed into the database. If any of the output data is missing then there has been an error. The user is then sent an email to alert them to this status change.

### Case-study: mapping malaria prevalence in Tanzania using data from the 2017 Malaria Indicator Survey

Data from the 2017 Malaria Indicator Survey (TMIS) was used to demonstrate the functionalities of Maplaria. The 2017 TMIS (a household survey) aimed to provide up-to-date estimates of basic demographic and health indicators related to malaria. It contains information on vector control interventions (mosquito nets, intermittent preventive treatment, and care seeking and treatment of fever in children) and testing of anemia and malaria infection in children aged 6–59 months. TMIS employed a two-stage sampling design based on the 2012 Population and Housing Census designed to produce representative estimates at the country level, urban and rural areas and across regions. In the first stage 442 clusters (127 urban and 315 in rural) consisting of enumeration areas (EAs) were selected with probability proportional to EA population size and with independent selection in each sampling stratum. All regions in Tanzania Mainland with a malaria prevalence below 10% were allocated 10 clusters (except for Dar es Salaam, which was allocated 15 clusters), areas with prevalence above 10% were allocated 20 clusters while Zanzibar regions were allocated 7 or 8 clusters each due to their small population size. In the second stage, 22 households per cluster were selected systematically from a household listing for a total of 9724 households (2793 in urban and 6931 in rural EAs).

All malaria data for tested children were then extracted. Testing was done through a drop of blood obtained via a finger (or heel) prick through rapid diagnostic test (RDT) after consent. The data was formatted for input to Maplaria and contained the variables described in Table 1.

**Fig. 2:**
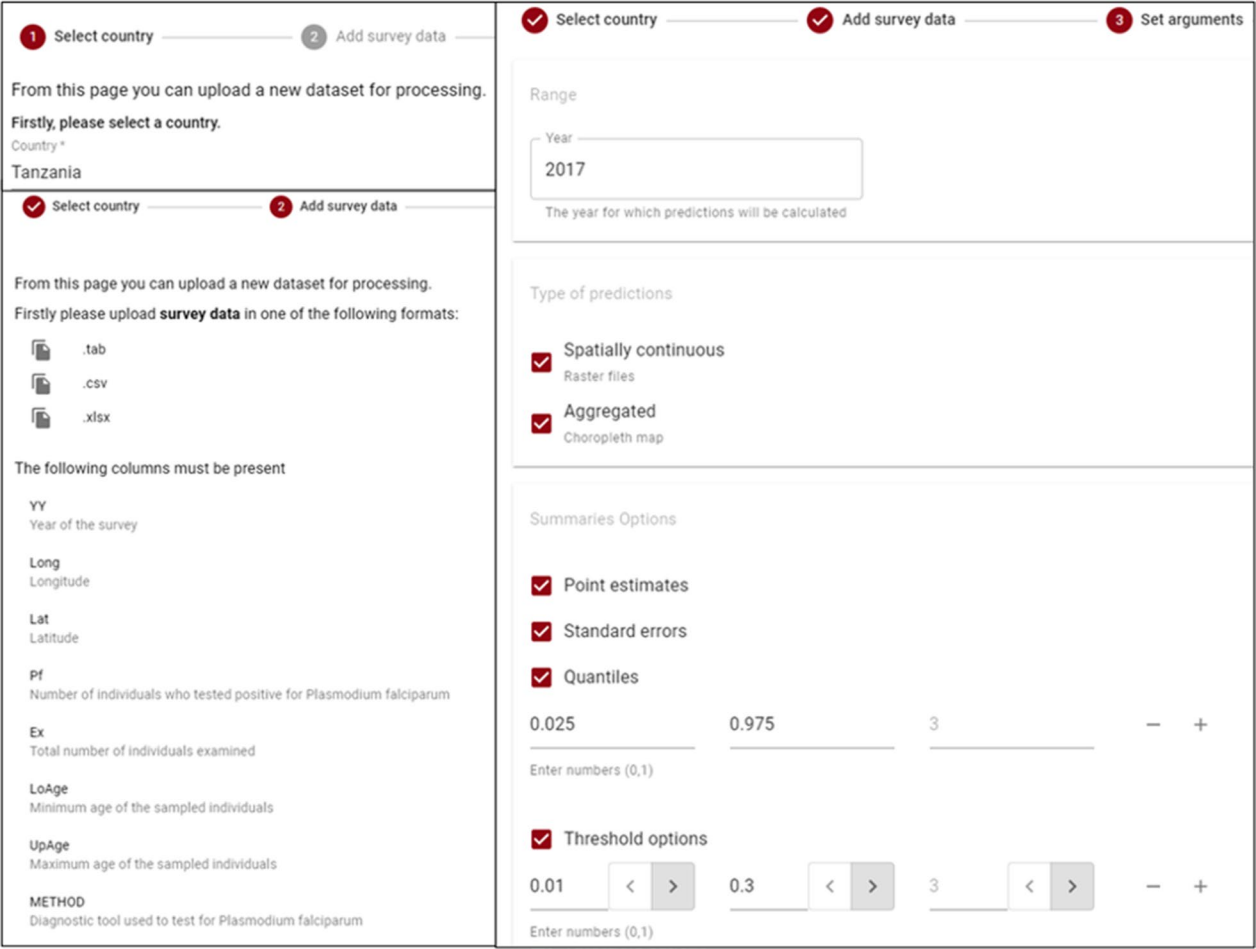
The first three steps in Maplaria; (1) selecting the country, (2) uploading survey data and 3) setting arguments

**Fig. 3:**
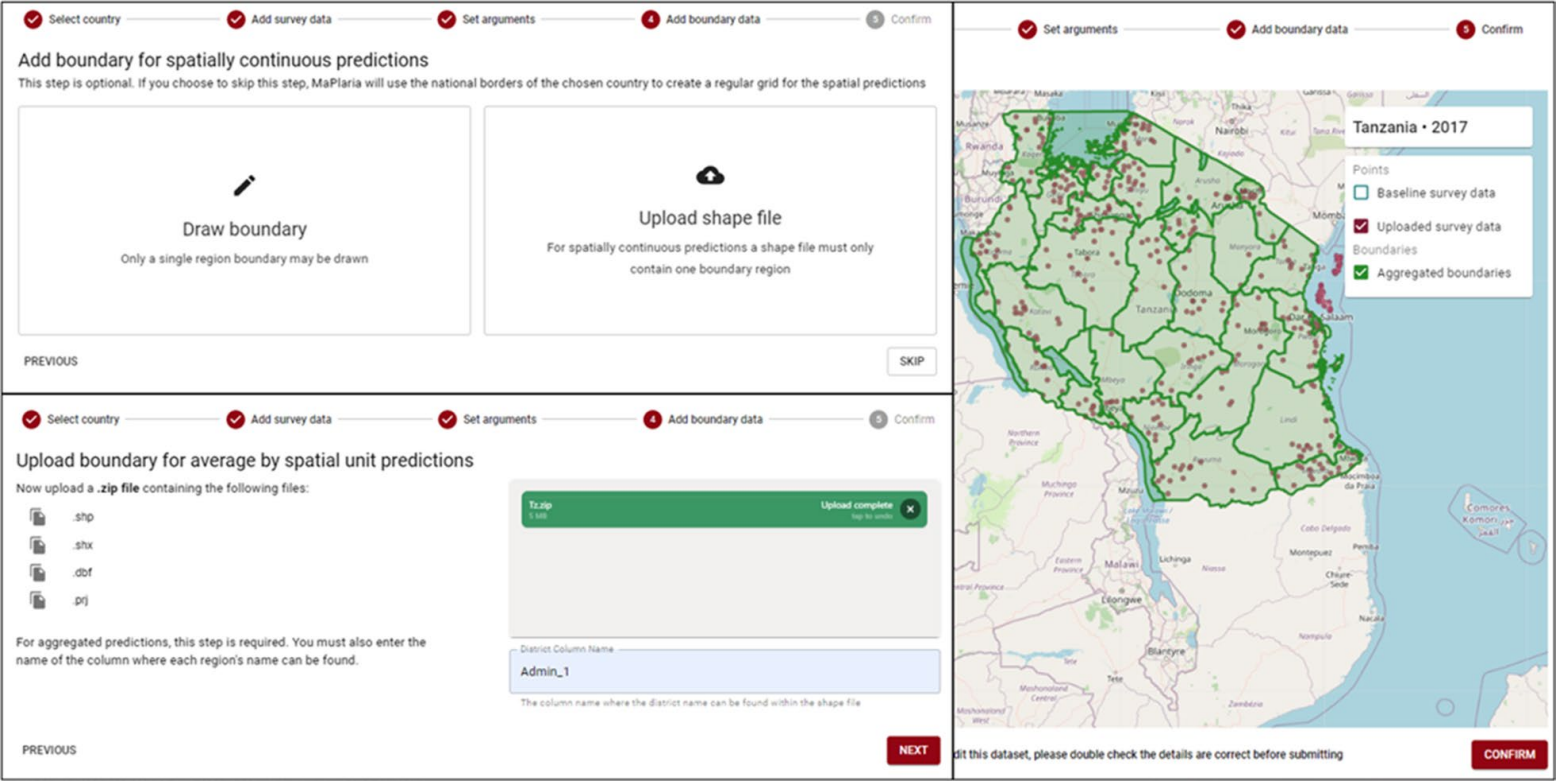
The fourth (adding or defining the boundary location) and fifth step (confirm all the sets and arguments) in Maplaria. An example for Tanzania 2017 MIS data

Figures 2 and 3 summarizes the process for uploading data onto Maplaria. In the first step, Tanzania was selected from a drop down menu of countries implemented in Maplaria which was followed by uploading a.csv file with the 2017 TMIS pre-formatted data. The model arguments were selected in the third stage, by setting 2017 as the prediction and opting for both aggregated and continuous point estimates with their corresponding standard errors, quantiles (0.025–0.97.5) and two threshold (1 and 30%) routinely used in malaria control [3].

**Fig. 4:**
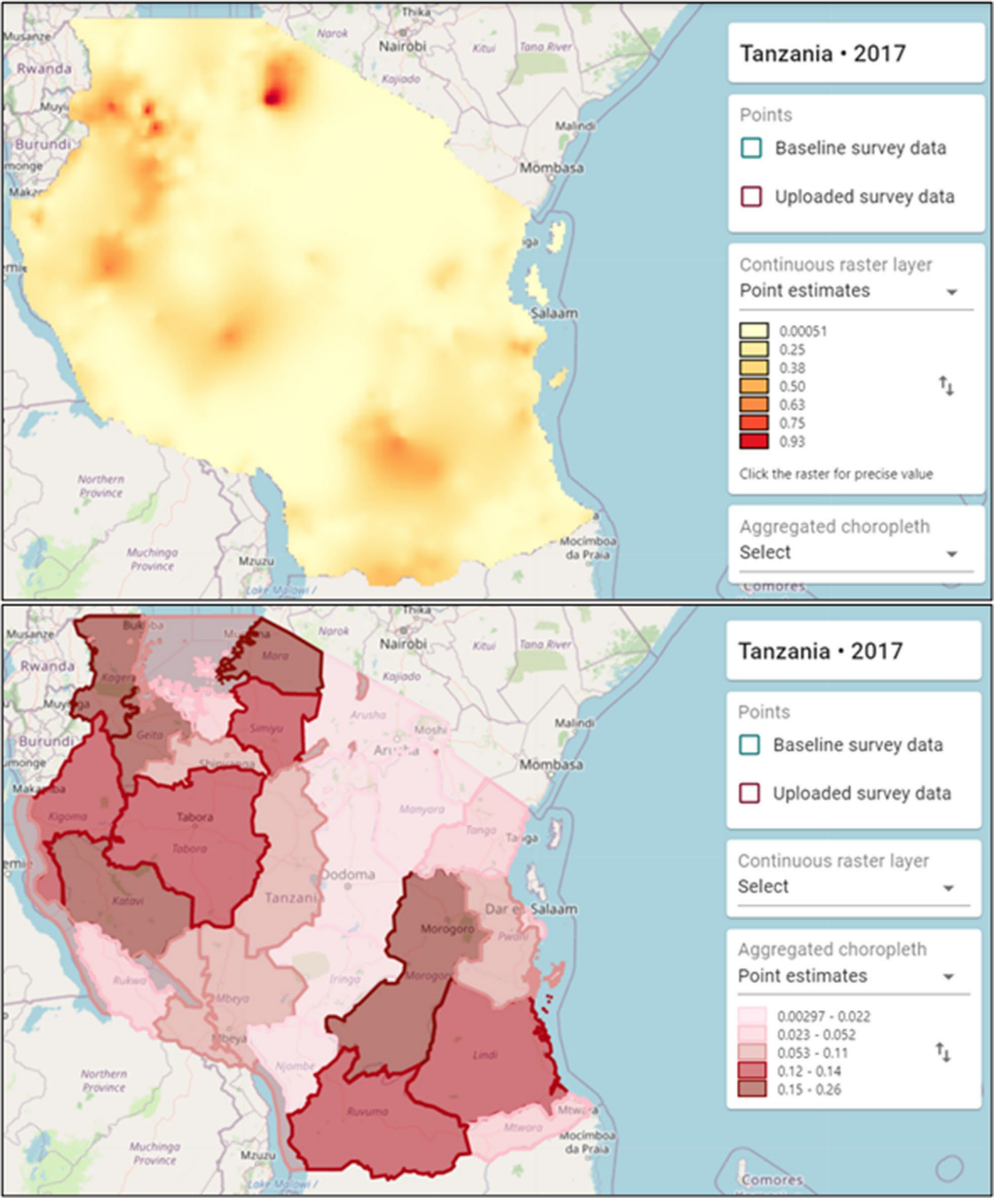
Examples of outputs in Maplaria showing continuous raster predictions and aggregated estimates within the districts. The outputs include all arguments that were set in step 3. All the estimates are available as a table and can be exported for use in other geospatial or statistical software

In the fourth stage, Tanzania subnational units were uploaded (Fig. 4). The subnational units were available at the Diva GIS data portal (https://www.diva-gis.org/) which provides global administrative boundaries by country. The subnational boundary was in a shapefile format which should have at least four files for Maplaria to execute with the following extensions: .shp (stores the feature geometry), .shx (stores the index of the feature geometry), .dbf (a database file used to store attribute data and object IDs), and .prj (stores the coordinate system information). The column holding the region name in the field “District Column Name'' was then specified. In the fifth step, the uploaded data and *baseline* data, were displayed on a basemap centred on Tanzania (Fig. 3). After confirming all the details, the work was submitted for processing.

The work status was monitored under the datasets menu, and an email notification (through the registered email) was received when the processing was completed. The results were available both on a pixel level (continuous) and aggregated level for the arguments that had been specified in the third stage. Figure 4 shows a sample of point predictions at two scales, both aggregated and continuous. All the outputs can be exported for use in other platforms and software. For example, maps of the predicted prevalence in raster format can be downloaded for further post-processing in open-source GIS software, where they can be combined with population density data to obtain estimates of the number of malaria cases at pixel-level.

## Discussion

The Maplaria web application offers a user-friendly interface for non-statistical users to carry out geostatistical prediction of annual malaria prevalence, by combining data that are uploaded by the user with existing publicly available data. The goal of Maplaria is to make advanced geostatistical methodology more accessible to NMCPs and provide information on the classification of evaluation units into the most likely endemicity levels. This is achieved by allowing the user to specify policy-relevant prevalence thresholds at any desired spatial scale. To circumvent the requirement for learning advanced statistical methods, the fitting and validation of the geostatistical models in Maplaria are carried out by geostatistical experts using existing publicly available data, whilst users are only tasked with specifying the options for carrying out the spatio-temporal predictions.

Decision makers in SSA have suggested that use of disease risk maps for strategic planning is dependent on the understanding of input data, data limitations, and how the data were used to derive the risk maps [10]. Maplaria is a useful tool that can help to maximize the use of MBG-generated disease risk maps by directly involving the end-user in the assembly and vetting of the input data, and set-up of the geostatistical predictions. This approach is likely to foster an increased sense of ownership of the generated malaria prevalence maps and improve the use of data for decision making in malaria endemic countries.

An important feature of Maplaria is that users can also carry out predictions using administrative boundaries and generate associated measures of uncertainties at different spatial scales. This is especially important in SSA countries where health officials use different administrative subdivisions for malaria planning decisions. For example, in Kenya, a national government health official will often require estimates for Kenya’s 47 subnational counties, while a county officer may require estimates at sub-county level or, in other cases, at ward level. In the presented example (Fig. 4), users can also make use of pixel level predictions to better understand the spatial variation in disease risk, whose pattern may be masked when considering aggregate predictions at the decision unit level. Furthermore, these risk maps can also be compared with historical malaria risk maps for monitoring, evaluation and receptivity, whilst exceedance maps can guide allocation of specific malaria control initiatives to specific areas.

One of the main limitations of Maplaria is that it can only accommodate a limited number of datasets to be processed in parallel, given the limited resources of a single server and hence the need for a queue. This can be improved in the future to allow Maplaria to run at scale. The processing of datasets could be disconnected from the API server, and the system could be horizontally scaled with multiple servers used for dataset processing in parallel. This could be deployed on a cloud computing platform to allow for elastic scaling based on user demand. An additional limitation is that Maplaria can only accommodate data-sets uploaded by the user that conform to the specific data-format of the *baseline* data from the Population Health Dataverse. However, despite all these limitations, Maplaria represents a first attempt in making advanced statistical methodology more accessible to non-statistical end-users and the framework illustrated in this paper can also be used to develop other user-friendly apps that could provide more flexible options in the set-up of the geostatistical predictions.

## Conclusions

The presented web-application, Maplaria, is a useful tool for making advanced geostatistical models more accessible to policy makers. In Maplaria, this is achieved by splitting the process of fitting and validating geostatistical models, carried out by a statistical expert, with the prediction stage, which is entirely user-driven. This framework can be used to develop more advanced web-applications that also allow for predictions of malaria prevalence at a finer temporal scale than Maplaria.

## Supplementary Information

**Additional file 1: Figure S1**. The uploading of data in Maplaria.

**Additional file 2: Figure S2**. The processing logic of data in Maplaria.

## Data Availability

The 2017 Tanzania malaria Indicator Survey (TMIS) data can be accessed by registering on the DHS portal while the baseline data can be publicly accessed at Harvard Data verse through https://dataverse.harvard.edu/dataset.xhtml?persistentId=doi:10.7910/DVN/Z29FR0.

## Abbreviations

API: Application Programming Interface
CI: Criteria one
C2: Criteria two
DHS: Demographic and Health Survey
EA: Enumeration area
Ex: Total children examined per EA
Lat: Latitude
Long: Longitude
LoAge: Lower age per EA
MBG: Model based geostatistics
MIS: Malaria Indicator Survey
NMCP: National Malaria control programme
*Pf*: Children who tested positive from the examined per EA
*Pf*PR_2-10_: *Plasmodium falciparum* Parasite rate standardised to the age group 2–10 years
RDT: Rapid diagnostic test
SSA: Sub Saharan Africa
WHO: World Health Organization
Upage: Upper age per EA
YY: Year
Pf: Children who tested positive from the examined per EA
