# Supplementary figures and images for: Maplaria: a user friendly web-application for spatio-temporal malaria prevalence mapping
Source: Malar J. 2021 Dec 20;20:471. doi: 10.1186/s12936-021-04011-7 (PMC8686323; doi:10.1186/s12936-021-04011-7)

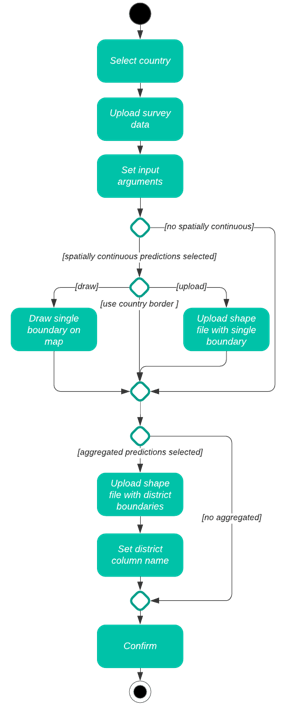

Supplement: Supplementary file 1 — Additional file 1: Figure S1. The uploading of data in Maplaria. [file 12936_2021_4011_MOESM1_ESM.tif]

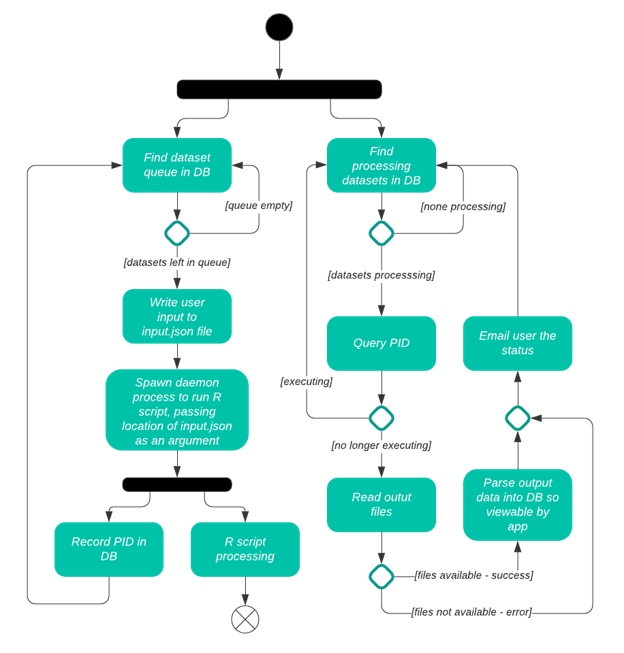

Supplement: Supplementary file 2 — Additional file 2: Figure S2. The processing logic of data in Maplaria. [file 12936_2021_4011_MOESM2_ESM.tif]
